# Jejunal adenocarcinoma; a case report and literature review

**DOI:** 10.1016/j.ijscr.2024.109372

**Published:** 2024-02-14

**Authors:** Hamed Tahmasbi, Parvin Kashani, Alireza Haghbin Toutounchi, Shaghayegh Sadeghmousavi, Arman Hasanzade, Mohammad Aghaei

**Affiliations:** a Department of General Surgery, Imam Hosein Medical and Educational Center, Shahid Beheshti University of Medical Sciences, Tehran, Iran; b Department of Emergency Medicine, Imam Hosein medical and Educational Center, Shahid Beheshti University of Medical Sciences, Tehran, Iran

**Keywords:** Small bowel carcinoma, Small bowel adenocarcinoma, Jejunal adenocarcinoma, Bowel obstruction, Intestinal obstruction

## Abstract

**Introduction and importance:**

Small bowel carcinoma (SBC) is a rare malignancy comprising mainly of adenocarcinoma and carcinoid tumors. Among SBCs, small bowel adenocarcinoma (SBA) accounts for 30–40 % and is predominantly found in the duodenum, while jejunal and ileal presence considered rare.

**Case presentation:**

We have presented a case of jejunal adenocarcinoma in a patient with obstruction symptoms. Prior to the obstruction, the patient mainly suffered from weakness and weight loss, in addition to iron deficiency anemia. During the investigation of underlying causes, we observed evidence of mass. However, before any additional evaluation could take place, the obstruction necessitated surgical intervention.

**Clinical discussion:**

Small bowel adenocarcinomas, particularly in the jejunum and ileum, are exceedingly rare and often present with complications such as obstruction, gastrointestinal bleeding, or perforation. Due to the non-specific symptoms, SBAs are challenging to diagnose before complications occur. SBAs are frequently diagnosed at advanced stages, so early diagnosis is crucial, as it can significantly impact patient survival. Thus, efforts should be made to expedite the diagnosis process to avoid complications and improve survival rates.

**Conclusion:**

SBAs are a rare condition, often diagnosed by related complications. Recognizing the importance of early diagnosis and its positive influence on patient survival, physicians and surgeons should consider SBA in patients presenting with relevant symptoms or cases of obstruction.

## Highlights

• Jejunal Adenocarcinoma poses diagnostic challenges due to its rarity.
• Delay in diagnosing jejunal adenocarcinoma may result in complications.
• A timely diagnosis significantly enhances the patient's prognosis.
• Physicians must be cautious and informed about this uncommon condition.

## 1 Introduction

The small bowel is the largest part of the gastrointestinal (GI) tract. Small bowel cancer (SBC) is a rare condition, accounting for only 3–5 % of all gastrointestinal malignancies. Adenocarcinoma includes 30–40 % of all SBCs [1,2]. In small bowel adenocarcinoma (SBA), the duodenum accounts for 57–60 %, the jejunum for 25–29 %, and the ileum for 10–13 % of cases [1,3]. Among SBAs, duodenal adenocarcinoma has the lowest survival rate, in contrast to jejunal adenocarcinoma, which exhibits a relatively better prognosis [4]. It is frequently detected in patients aged of 60 years and nearly 80 % of cases are diagnosed at the stage of III or IV, often presenting with complication, such as obstruction or bleeding [3]. This neoplasm is recognized as a rare cause of small bowel complications such as obstruction [5]. We have presented a case of jejunal adenocarcinoma. The purpose of this manuscript is to highlight the importance of the timely diagnosis of this tumor which is often misdiagnosed. This article has been reported in line with the SCARE 2023 [6].

## 2 Presentation of case

### 2.1 History

A 49-years-old male patient was presented to emergency department suspected to obstruction. The patient had a recent admission with complaints of weight loss of 15 kg over the past 4 months, weakness, chronic fatigue, anorexia, and nocturnal hyperhidrosis. Upon evaluation, lab tests showed an iron deficiency anemia, then endoscopy and colonoscopy were performed without any remarkable finding. Following the push enteroscopy, a large circumferential, fungating, infiltrative and ulcerated mass was seen in proximal part of jejunum and biopsy was taken from the lesion. Pathology reported a well-differentiated invasive adenocarcinoma. The patient was scheduled for double balloon endoscopy and MR Enterography to conduct further evaluation and consultant make decision about possible chemotherapy. However, he readmitted emergent with complaints of severe abdominal pain and biliary vomiting from past day. He mentioned no defecation in past 2 days. He had a history of consuming alcohol but no smoking. Also, he did not mention any familial history of malignancy.

### 2.2 Assessment

**Fig. 1:**
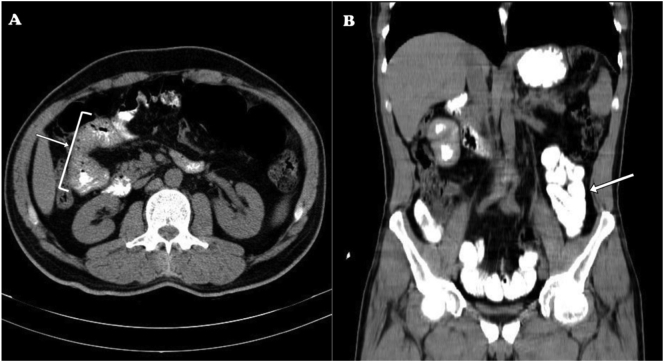
Patient's contrast-enhanced abdominopelvic CT scan, axial view (A) and coronal view (B); arrows indicate contrast-highlighted intestinal dilation filled with food impaction, suggesting the presence of a bowel obstruction.

On physical examination, an abdominal tenderness was positive mostly in epigastric region. Rebound was negative. Bowel sound was reduced. Abdominopelvic CT scan with contrast enhancement revealed partial obstruction and food impaction in proximal of jejunum, in addition to thickening of intestinal wall over 13 mm (Fig. 1). Also, enhanced adjacent reacted mesenteric lymph nodes were detected in area with SAD = 12 mm. Finally, the patient underwent an emergency surgery due to small intestine obstruction.

### 2.3 Operation summary

**Fig. 2:**
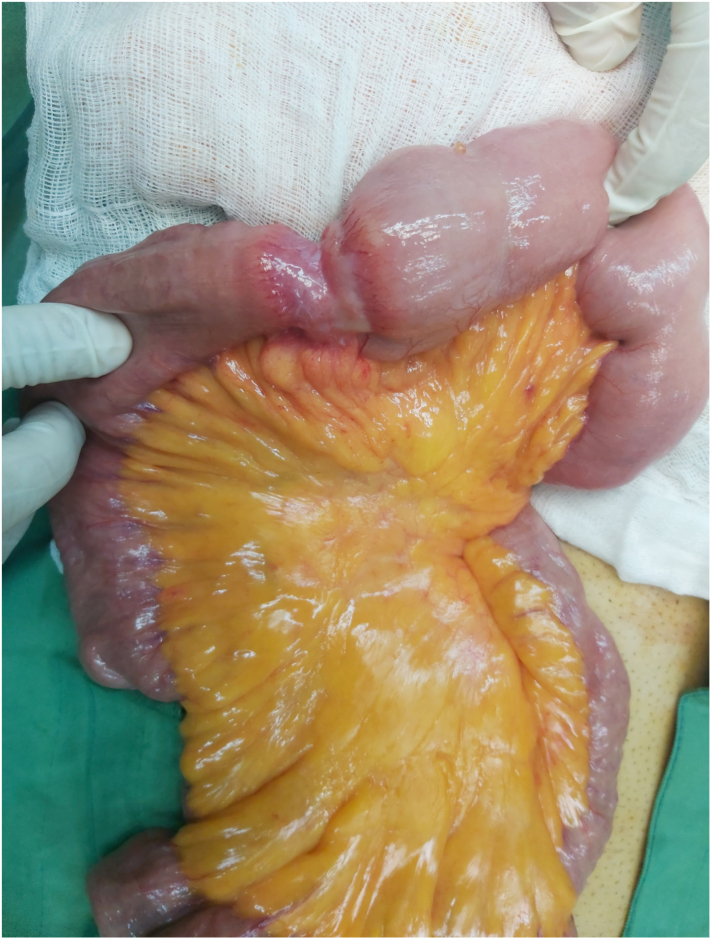
Obstructive lesion at the proximal of the Jejunum.

An emergency laparotomy under general anesthesia through an upper midline incision was performed. Following the exploration, a 3 cm thick mass was observed approximately in 20 cm of the Treitz ligament (Fig. 2). A segmental resection about 20 cm of jejunum was done and safe margin conformed by frozen simultaneously. Then side to side early anastomosis was performed by stapler. Whole of peritoneal cavity was explored without any omental, peritoneal, or liver suspected lesion.

### 2.4 Outcome

Following the surgical resection, a pathological evaluation of the excised mass confirmed the presence of malignant mass along with 15 adjacent mesenteric lymph nodes. Pathology reported well-differentiated invasive adenocarcinoma (G1) invading through the muscularis propria into the peripheral adipose tissue (T3). All surgical margins were free of tumor. Lymphovascular invasion was identified, while Perineural invasion was not identified. Fifteen lymph nodes were submitted‚ all of them were free of tumor (N0). Overall pathologic staging was PT3NOMx. In addition, IHC study resulted in; Synaptophysin: Negative and Chromogranin: Negative. Reassuring to find that the surgical margin and all lymph nodes were clear of tumors, indicating that the cancer had not spread beyond the localized area. After a smooth recovery without any complications, the patient was referred to an oncologist with the diagnosis of jejunal invasive adenocarcinoma for a comprehensive treatment plan. The adjuvant chemotherapy has been followed by FOLFOX (5-fluorouracil and oxaliplatin). During 1-year follow-up until now, the patient remained asymptomatic and did not report any completion related to surgery and chemotherapy.

## 3 Discussion

Small bowel carcinoma (SBC) is a rare malignancy, accounting for approximately 5 % of all neoplasms within the GI tract. The estimated annual prevalence is 0.3–2.0 cases in every 100,000 persons and the most incidence in the African population, and recently, there has been an upward trend in its occurrence [1]. The prevalence of SBC is higher after the age of 40, with the most cases being diagnosed among patients aged 55–64. Currently, five-year survival rate in the USA is 65.5 %, while the stage of cancer at diagnosis significantly impacts survival [7].

SBC is classified into four prevalent histological types including carcinoid tumor (35–42 %), adenocarcinoma (30–40 %), lymphoma (15–20 %), and sarcoma (10–15 %) respectively [8]. While SBAs appear to have a greater tendency to develop in the microenvironment of the duodenum, jejunum involvements are rarer [9]. The higher levels of IgA and the rapid transit of contents through the small bowel compared to the large bowel in addition to the lower bacterial load in the small bowel and its higher sensitivity to stress may contribute to the reduced prevalence of small bowel neoplasms are mentioned for explaining the lower rate of small bowel malignancies [10]. Various factors have been associated with the incidence of SBA, including alcohol consumption, smoking, obesity, diet, and chronic inflammatory conditions such as Crohn's disease, celiac disease, and cystic fibrosis. Moreover, specific hereditary cancer syndromes, such as hereditary non-polyposis colorectal cancer (HNPCC), Peutz-Jeghers, and familial adenomatous polyposis (FAP), are also linked to an increased risk of SBA [1,2,3,4]. SBAs are commonly diagnosed when malignancy had progressed to the late stages (III and IV) due to nonspecific symptoms. As a result, SBA is often found whenever complications such as obstruction, perforation or bleeding happen. Generally, non-specific symptoms associated with SBA lead to an average delay of 6–10 months in the diagnosis [11].

CT scan is commonly used as a preferred diagnostic method in patients with GI symptoms. However, its accuracy in detecting tumors via this modality has been reported to be around 50 %. Moreover, CT scan lacks detailed information about the intestinal mucosa and may miss smaller and flatter lesions. Consequently, choosing the most suitable imaging tool for diagnosis is often challenging. As a result, many cases are diagnosed intra-operatively [12,13]. Barium swallow is suggested to have a sensitivity of up to 50 % in detecting small bowel tumors. Endoscopic techniques have limited capability in fully visualizing the small bowel. Thus, double-balloon and push enteroscopy have a better chance to explore more distal parts of the intestinal tract, but these are more available in advanced medical centers [12]. Therefore, the diagnosis of small bowel pathologies remains challenging.

Li et al. presented a case of a 26-year-old man with SBA in the jejunum, who initially had no specific symptoms. They ultimately diagnosed SBA through a combination of balloon-assisted enteroscopy, positron emission CT scans, and the measurements of carcino-embryonic antigen (CEA) and carbohydrate antigen (CA) 19-9. The patient underwent segmental intestine with lymph node resection followed by palliative chemotherapy [7]. Notably, the significant aspect of this case was that the patient was younger in contrast to typical SBA patients. However, the diagnosis was also delayed. Another case report described a patient with nonspecific symptoms who underwent explorative laparotomy, revealing a jejunal stricture and dilated proximal small bowel loops. Histopathology and metastatic investigation confirmed moderately-differentiated adenocarcinoma of the jejunum [5]. These cases highlight the importance of considering jejunal adenocarcinoma as a possible differential diagnosis when encountering patients with symptoms and signs of small bowel obstruction, particularly due to the common occurrence of delayed diagnosis or misdiagnosis in this type of cancer, leading to complications. In a case series by Manley et al., four cases of SBA were presented. One patient presented with iron-deficient anemia similar to the recent case, and three other patients presented with obstruction. Prompt diagnosis was achieved for the three obstruction cases, whereas the patient with anemia required further investigations before a diagnosis was reached. The authors concluded that SBA should be highly suspected in cases presenting with anemia due to occult gastrointestinal bleeding, and appropriate investigations should be promptly requested [9]. These case presentations underscore the importance of early consideration and investigation of SBA in patients with suggestive symptoms to avoid potential delays in diagnosis and improve patient outcomes. According to the latest evidence, seems already a surgical resection with safe and clear margins is the only curative option, but Zaanan et al. Stated that adjuvant chemotherapy adds a significant survival benefit in patients with stage II and III. They also indicated the primary tumor location as a predictive factor of response to adjuvant chemotherapy [14].

## 4 Conclusion

We presented a case of SBA initially presented with iron deficiency anemia. The delay in diagnosing eventually led to small bowel obstruction. We conclude that although the incidence of SBA is low, physicians should consider it as a potential cause of small bowel obstruction. Due to the challenges in accessing and diagnosing these tumors, different diagnostic tools such as tumor markers should be employed for further investigations in these patients.

## Consent

Written informed consent was obtained from the patient for publication of this case report and accompanying images. A copy of the written consent is available for review by the Editor-in-Chief of this journal on request.

## Ethical approval

At our institution, we have a specific policy regarding ethical approval for deidentified case reports. In such cases, where patient identification and sensitive information have been removed, the requirement for ethical approval is waived. This policy is overseen by the Research Ethics Committee of School of Medicine – Shahid Beheshti University of Medical Sciences.

## Funding

This article did not receive funds.

## Author contribution

Conceptualization and Methodology: Dr. Hamed Tahmasbi, Dr. Parvin Kashani

Writing first draft: Dr. Shaghayegh Sadeghmousavi, Dr. Arman Hasanzade

Investigation: Dr. Alireza Haghbin Toutounchi, Dr. Arman Hasanzade

Data curation: Dr. Mohammad Aghaei, Dr. Alireza Haghbin Toutounchi

Writing–review & editing: Dr. Alireza Haghbin Toutounchi

All authors read and approved the final manuscript

## Guarantor

Dr. Mohammad Aghaei.

## Conflict of interest statement

All authors declare that they have no conflicts of interest.
